# Rubber Dam Holder

**Published:** 1881-12

**Authors:** 


### RUBBER DAM HOLDER.

When the rubber dam is applied in the usual manner to the teeth, it is in every instance desirable, and in some indispensable that the free flap projecting over the chin, should be drawn down sufficiently to be out of the way, and prevent wrinkling and puckering in the mouth. The common method of doing this has been by the use of weights, with hooks or clasps attached by means of which they are fastened to the rubber, these have never been entirely satisfactory. The traction of corners is always directly downward from the point of attachment, except by the use of some complicated adjustment.

After the expenditure of an immense amount of time, expense, effort, and inventive talent! It has been ascertained that this object can be obtained by the use of little elastic clasp or catch bands, such as are sometimes worn to hold up the shirt sleeves. They are elastic bands from three to four inches long, with a little spring clamp on each end. The nipping edges of the clamps should be serrated, or rather get those which are serrated; they can be obtained at almost any notion or gentlemen's furnishing store, for the sum of ten cents per pair.

By the attachment of these to one end of the rubber, and the other to an edge or fold of the garment below, a most perfect adjustment can be made. By cutting this elastic in two in the middle, and sewing in a sufficient length of the common elastic band, an excellent holder for the upper corners of the rubber is made. From four to six of these bands of various lengths are very useful for holding, suspending, attaching, etc.

Try them.

